# Assessment of Mobile Health Apps Using Built-In Smartphone Sensors for Diagnosis and Treatment: Systematic Survey of Apps Listed in International Curated Health App Libraries

**DOI:** 10.2196/16741

**Published:** 2020-02-03

**Authors:** Clarence Baxter, Julie-Anne Carroll, Brendan Keogh, Corneel Vandelanotte

**Affiliations:** 1 School of Public Health and Social Work Faculty of Health Queensland University of Technology Kelvin Grove, Queensland Australia; 2 Institute of Health and Biomedical Innovation Queensland University of Technology Kelvin Grove, Queensland Australia; 3 Digital Media Research Centre Creative Industries Faculty Queensland University of Technology Kelvin Grove, Queensland Australia; 4 School of Health, Medical and Applied Sciences Central Queensland University Rockhampton, Queensland Australia

**Keywords:** telehealth, mHealth, smartphone, mobile apps, instrumentation, health care quality, health care access, and health care evaluation, medical informatics, consumer health informatics, physician-patient relations, prescriptions, patient participation, patient-generated health data, diagnostic self evaluation, self-care, self-management, medical device legislation

## Abstract

**Background:**

More than a million health and well-being apps are available from the Apple and Google app stores. Some apps use built-in mobile phone sensors to generate health data. Clinicians and patients can find information regarding safe and effective mobile health (mHealth) apps in third party–curated mHealth app libraries.

**Objective:**

These independent Web-based repositories guide app selection from *trusted* lists, but do they offer apps using ubiquitous, low-cost smartphone sensors to improve health? This study aimed to identify the types of built-in mobile phone sensors used in apps listed on curated health app libraries, the range of health conditions these apps address, and the cross-platform availability of the apps.

**Methods:**

This systematic survey reviewed three such repositories (National Health Service Apps Library, AppScript, and MyHealthApps), assessing the availability of apps using built-in mobile phone sensors for the diagnosis or treatment of health conditions.

**Results:**

A total of 18 such apps were identified and included in this survey, representing 1.1% (8/699) to 3% (2/76) of all apps offered by the respective libraries examined. About one-third (7/18, 39%) of the identified apps offered cross-platform Apple and Android versions, with a further 50% (9/18) only dedicated to Apple and 11% (2/18) to Android. About one-fourth (4/18, 22%) of the identified apps offered dedicated diagnostic functions, with a majority featuring therapeutic (9/18, 50%) or combined functionality (5/18, 28%). Cameras, touch screens, and microphones were the most frequently used built-in sensors. Health concerns addressed by these apps included respiratory, dermatological, neurological, and anxiety conditions.

**Conclusions:**

Diligent mHealth app library curation, medical device regulation constraints, and cross-platform differences in mobile phone sensor architectures may all contribute to the observed limited availability of mHealth apps using built-in phone sensors in curated mHealth app libraries. However, more efforts are needed to increase the number of such apps on curated lists, as they offer easily accessible low-cost options to assist people in managing clinical conditions.

## Introduction

### Background

With origins in the early 1990s and the inception of devices such as the IBM *Simon* Personal Communicator, early smartphone devices offered *untethered* mobile telephony, augmented by a humble suite of modular apps to extend basic phone functionality, hence the *smart* moniker in *smartphone* [1,2]. Nearly 3 billion people worldwide now use smartphones [3]. The release of the Apple iPhone in 2007 and subsequent competing Android smartphone offerings from Google and other vendors saw the emergence of platform-specific app stores, offering downloadable apps for a myriad of purposes [4]. Of the estimated 4.5 million apps available in the Google and Apple app stores, a million collectively pertain to health, fitness, nutrition, and well-being in general [5,6]. A subset of 300,000 of these apps may be regarded as *bona fide* mobile health (mHealth) apps, some of which may be potentially prescribed to patients for the diagnosis or treatment of health conditions [7]. Acknowledged by the World Health Organization in 2011, mHealth is defined as medical and public health practices supported by mobile devices, such as mobile phones, patient-monitoring devices, personal digital assistants, and other devices such as wearables [8]. More than 500 million people worldwide are believed to have downloaded one or more mHealth apps to their mobile phone [9].

#### Digital Disruption, Prescription, and Self-Prescription of Apps

Innovation is a hallmark of developments in medical technology, with a rich pedigree that long precedes contemporary *digital disruption* such as that attributable to mobile telephones and related technologies [10]. In 1995, Christensen and Bower [11,12] coined the term *disruptive technology* (later termed *disruptive innovation*) to describe the creation of new markets in response to novel emergent technologies based on values that are different from that of existing markets. Ubiquity, accessibility, and familiarity with mobile phone technology, combined with increasing general interest in health and the rising cost of clinician-led health care, may all contribute to the emergence of one such new *disrupted* market, namely, in the context of mHealth [10]. Health consumers may now independently seek out mHealth apps to assist with the diagnosis or management of health conditions [13]. Mobile phone camera apps for wound care and microphone apps for sleep apnea management are examples of mHealth apps using built-in sensors where diagnostic or treatment procedures once restricted to the realms of formal medical consultation are now accessible to laypersons for download and *self-prescription*, constituting potential disruption, which circumvents traditional clinician-initiated care and supervision [14,15].

Self-management of health conditions without adequate medical guidance (colloquially termed the *Dr Google* effect) is viewed by some as a *disruption* to traditional doctor-patient relationships, with potential risks of delayed (or incorrect) diagnosis or inadequate treatment because of the selection of malfunctioning and ineffective or inappropriate mHealth apps [16,17]. On the contrary, others cite the emergence of the *Quantified Self* movement in the 1970s and ensuing developments in areas such as Precision Medicine as offering patients the opportunity to leverage mobile phone technology to improve health, heralding a *democratization of information control* in health care [18-21]. In contrast to self-initiated engagement with mHealth, some apps may be prescribed to patients under the guidance of health professionals [7,15]. *Badly behaving* mHealth apps pose regulatory challenges regarding the evidence of app quality, safety, and efficacy and present risks to human health by potential misdiagnosis and inadequate or ineffective treatments, or by delaying face-to-face medical consultations [7,15,22,23].

#### Taxonomies for Mobile Health App Sensors

Several taxonomies exist for describing mHealth apps; one simple method categorizes them as either passive or active [23]. Passive mHealth apps display static health information pages or acquire hand-keyed input of health information. In contrast, active mHealth apps generate some form of health data [13,23]. It is in this latter active realm that sensor-based mHealth apps reside. Built-in smartphone sensors are readily accessible in the devices owned by billions of mobile phone users worldwide. Smartphones have evolved to incorporate environment and position sensors to augment and enhance device functionality [24]. In addition to sound detection by the phone microphone, cameras document the visual world [25]. Touch screens facilitate flexible display presentation and command initiation [25]. Accelerometers sense device orientation and adjust screen display layout in either portrait or landscape modes accordingly, whereas GPS locates devices geographically [25].

#### Regulation and Compliance Issues

The utility of such a trove of sensors has not gone unnoticed by clinicians and app developers alike [24,26]. Pedometer apps have been coded to count steps based on accelerometer monitoring [27]. Photoplethysmography apps leverage mobile phone cameras to detect changes in skin color with blood flow, estimating respiratory rate, heart rate (and heart rate variability), blood pressure, and blood oxygen saturation [28-31]. Examples abound as to innovative uses of sensor information for gathering health data [26]. In contrast, examples also exist highlighting deficiencies in some sensor-based mHealth apps. For example, blood pressure values based on pulse estimates from a particular camera-based smartphone app were demonstrated to be erroneous, potentially exposing hypertensive persons to harm with spurious readings [32]. Oximetry readings from another camera-based app were found to be inaccurate, with the potential for incorrect blood oxygen saturation readings to put users at risk [33]. Regulatory authorities worldwide seek to mitigate this risk by deeming any app that attempts diagnosis or treatment to be a medical device, requiring rigorous evaluation, testing, and regulatory control [4]. Given the impost this places on app developers, some have sought to circumvent regulation by defining some apps as for entertainment or recreation or by using sensor-generated data as an adjunct to an app’s operation as opposed to its main purpose [23,34].

#### Searching for Apps Using Curated Libraries

App stores such as those offered by Apple and Google present literally millions of results in response to searches on health topics [4,35]. A 2016 review of clinical and health care–related apps in the Google and Apple app stores found 36 apps for clinical diagnosis and 44 patient health monitoring apps, with the mobile phone camera identified as the predominant built-in sensor used [35]. Mobile phone camera apps offered for image-based diagnosis of eye and skin conditions, or photoplethysmographic monitoring of pulse and estimated blood pressure, and sleep apnea diagnostic apps using mobile phone microphones are examples of sensor-based apps offered by major app stores [15,34,35]. Information regarding vetting procedures for the inclusion of apps in these vendor stores is not publicly available [36]. For example, Apple is reported to have introduced additional requirements for developers regarding the measurement accuracy of apps, but details of these requirements remain undisclosed [37]. The quality and safety of mHealth apps offered by these vast stores are questioned by some, as is the utility of listed app descriptions in facilitating informed use of apps in a prescription context [37,38]. App listing and availability are also tempered by emergent government medical device regulatory requirements in Europe, the United States, and elsewhere [4,36,39].

Distinct from app stores such as the Apple App Store and Google Play Store, a number of independent third-party mHealth app repositories have emerged, with the intent of providing curated *trusted* lists of health apps for users to review and to guide the selection of safe and effective mHealth apps [39]. Also known as health app clearinghouse websites, these libraries are Web-based portals that do not host apps per se but offer information and links to a range of vetted apps that have satisfied selection criteria required for inclusion in the respective repository [40-42]. Examples of such libraries include the government-funded National Health Service (NHS) Apps Library in the United Kingdom and two privately funded repositories, namely, AppScript in the United States and MyHealthApps in Europe and the United Kingdom [43]. Curation of apps submitted to these libraries consists of varying degrees of scrutiny [39]. Submissions to the NHS Apps Library and AppScript repositories require app developers to respond to questions regarding app quality and safety, which are evaluated by curators of these libraries using proprietary scoring methodologies, whereas the MyHealthApps site incorporates reviews from patients [13,22,44]. Intended audiences for such curated apps include clinicians (with the intent to prescribe an app for use by a patient) as well as laypersons seeking to self-manage their health. In contrast to reviews regarding the availability of mHealth apps in popular Google and Apple app stores (including those using built-in smartphone sensors), there is a paucity of information regarding sensor-based mHealth apps offered by third party–curated mHealth app libraries [4,16,35].

### Objective

Given the potential for health improvement arising from the availability and utility of built-in sensors in billions of smartphones worldwide, the purpose of this systematic survey was to identify smartphone mHealth apps using built-in sensors, offered by three popular contemporary international curated mHealth app repositories, and to assess which health conditions these apps address and whether they are available across different platforms [39,43,45].

## Methods

### Libraries Selected for Survey

**Figure 1:**
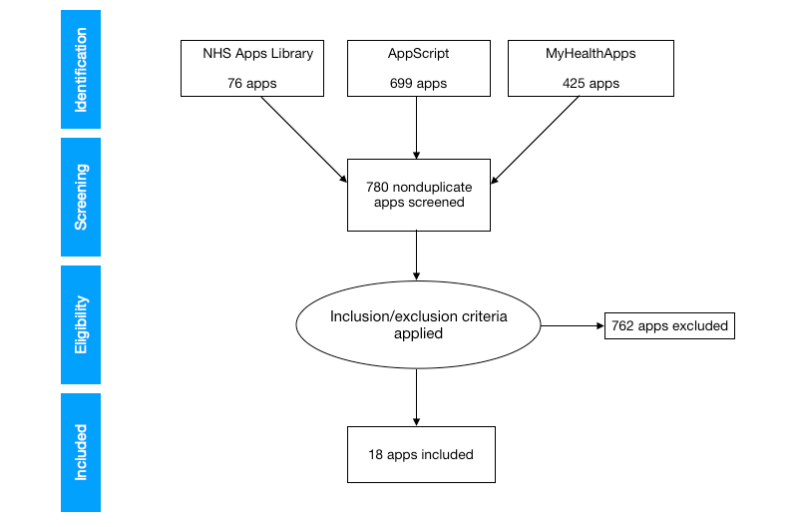
Preferred Reporting Items for Systematic Reviews and Meta-Analyses diagram for survey.

This survey, conducted in October 2019, considered all mHealth app listings in the NHS Apps Library, AppScript, and MyHealthApps–curated mHealth app repositories (Figure 1) [46-48]. These libraries were selected as examples of government-funded (NHS Apps Library) and privately funded curated mHealth app repositories (AppScript and MyHealthApps) [4,15,36,41]. The latter two privately funded libraries differ in that MyHealthApps incorporates patient reviews in the curation process, whereas AppScript uses a proprietary scoring process [22,41,44].

### Identification of Apps for Inclusion

All apps addressing health conditions using built-in mobile phone sensors to generate health data were identified using the publicly accessible search interfaces offered by each repository. As no search criteria were offered by these sites for filtering and identifying sensor-based apps, manual screening of the descriptions of all individual apps listed by each website was conducted by the lead researcher to screen for the use of built-in mobile phone sensors. Curated library descriptions for identified apps were inspected to categorize the purpose of the app as solely diagnostic, therapeutic, or a combination of both. Diagnostic apps were defined as those that identify the nature of a health condition, in contrast to treatment apps, which offered features for health condition management. Health conditions addressed by included apps were classified based on the description provided by each repository.

### Exclusion Criteria

Apps using external or add-on sensors and nonsmartphone wearable device apps were excluded from this survey, as external components may impose additional cost, complexity, or excessive battery consumption, potentially reducing availability or accessibility to smartphone mHealth users [49]. *Propeller* is an example of an asthma therapy coaching app using an external Bluetooth sensor, which was excluded from this study [47]. Exercise, general activity, and accessibility apps were also excluded, as only apps used in diagnosis or treatment of specific health conditions were in scope of this survey. *Runkeeper* is an example of a GPS running tracker designed for monitoring exercise but excluded from this study, as no specific health condition was indicated for its use [47].

### Availability of Apps

Mobile phone operating systems supported by included apps were noted, assessing the availability of these apps by users of the Apple iOS and Google Android phone types. The availability of included apps on advertised platforms was confirmed by following links advertised by each repository to inspect the Apple and Google app store app listings for apps. Apps were not downloaded or tested. App listings included as in scope by the lead researcher were then reviewed by the research team.

Low numbers across result groups precluded rigorous statistical analysis. Descriptive statistics were used where appropriate to illustrate results and to allow comparison between different libraries proportionate to respective library size.

## Results

### Overall Findings

A total of 1200 apps listed in the three selected curated mHealth app repositories were identified (Figure 1). Of 1200 apps, 780 nonduplicated apps were screened for eligibility. A total of 18 mHealth apps using built-in smartphone sensors were found in the three repositories surveyed. These represented 1.1% (8/699) to 3% (2/76) of the total app count across respective curated libraries (Table 1).

**Table 1:** Built-in sensor smartphone apps found in surveyed mobile health app libraries.

Curated mobile health app library	Total apps identified (n=1200), n	Built-in sensor apps included (n=18), n (%)
NHS^a^ Apps Library	76	2 (3)
AppScript	699	8 (1.1)
MyHealthApps	425	8 (1.9)

^a^NHS: National Health Service.

### Included Apps

Details of smartphone mHealth apps using built-in sensors included from each respective curated mHealth library in this survey are presented in Multimedia Appendix 1. Listings include app type (ie, diagnostic, therapeutic, or both), sensor type used, app name, description, mobile phone operating system, and health concern addressed by each app.

### Cross-Platform Availability

Half (9/18, 50%) of all apps inspected were offered solely for use on the Apple iOS platform, with a further 11% (2/18) dedicated to the Android operating system (Table 2). Only about one-third (7/18, 39%) of the identified apps across all surveyed libraries were available for cross-platform use on Apple iOS and Android operating systems. The MyHealthApps repository offered the greatest cross-platform app availability, with 5 of the 8 (63%) identified apps in this library compatible with Apple iOS and Android operating systems. Most AppScript listings identified (6/8, 75%) were compatible only with Apple iOS.

**Table 2:** Operating systems for apps using built-in mobile phone sensors.

Operating system	Curated mobile health app library			Total, n (%)
	NHS^a^ Apps Library	AppScript	MyHealthApps	
Apple iOS only	1	6	2	9 (50)
Android only	0	1	1	2 (11)
Both	1	1	5	7 (39)
Total	2	8	8	18 (100)

^a^NHS: National Health Service.

### Purpose of the Apps

Almost one-fourth (4/18, 22%) of all included apps were dedicated entirely to the diagnosis of health conditions (predominantly available in MyHealthApps), whereas half were solely treatment oriented (Table 3). The AppScript and MyHealthApps libraries offered comparable numbers of combined diagnostic and therapeutic apps using built-in sensors, whereas more apps dedicated to treatment were available in AppScript compared with the other libraries.

**Table 3:** Purpose for mobile health apps identified using built-in mobile phone sensors.

Purpose	Curated mobile health app library			Total (n=18), n (%)
	NHS^a^ Apps Library (n=2)	AppScript (n=8)	MyHealthApps (n=8)	
Diagnostic (Dx)	0	1	3	4 (22)
Therapeutic (Rx)	2	5	2	9 (50)
Both	0	2	3	5 (28)

^a^NHS: National Health Service.

### Mobile Phone Sensors Used

Camera (7/18, 39%) and touch screens (6/18, 33%) were the most frequently identified smartphone sensors used (Table 4). Microphones and accelerometers (and mobile phone speakers) were found to be less frequently used sensors in the identified apps. No GPS-based mHealth apps were identified in this survey. MyHealthApps offered more camera-based apps than the other libraries combined, whereas AppScript listed more apps using touch screens and microphones.

Smartphone cameras assessed pulse rate using photoplethysmography in an anxiety treatment app (Beat Panic), a respiratory therapy app (HeartRate+ Coherence), and a cardiac app (Instant Heart Rate). Beat Panic and Heart Rate+ Coherence are examples where smartphone pulse rate sensing is a secondary function to support a main therapy, namely, anxiety management and breathing exercise, respectively. Camera images were also used for automated skin cancer diagnosis (SpotMole) and in capturing images for dermatological diagnosis (UMSkinCheck, iDoc24, and MyPso).

In addition to capturing responses to speaker-generated tones in audiology testing, touch screens were used in vision training (Vision training 1 and Visual Attention Therapy Lite), neurological tremor assessment (pdFIT and Dexteria), and anxiety management (Chill Panda and Antistress Chromotherapy). Microphone sensors were used in several respiratory therapy apps (Breathing Zone, SnoreLab, and SnoreMonitor SleepLab). The use of a mobile phone accelerometer sensor was identified in a single app for neurological tremor assessment (LiftPulse).

**Table 4:** Sensor types found in curated mobile health app libraries.

Sensor	Curated mobile health app library			Total (n=18), n (%)
	NHS^a^ Apps Library (n=2)	AppScript (n=8)	MyHealthApps (n=8)	
Camera	1	2	4	7 (39)
Touch screen	1	3	2	6 (33)
Microphone	0	3	0	3 (17)
Accelerometer	0	0	1	1 (6)
Speaker	0	0	1	1 (6)

^a^NHS: National Health Service.

### Health Conditions Addressed

Respiratory (4/18, 22%), dermatological (4/18, 22%), neurological (3/18, 17%), anxiety (3/18, 17%), and visual health (2/18, 11%) were the predominant health concerns addressed by the identified apps (Table 5). MyHealthApps and AppScript libraries listed more apps addressing a wider range of health conditions than the NHS Apps Library. The AppScript repository presented more apps for respiratory-related conditions, concerning snoring (n=2) and breathing exercises (n=2). Both the apps using built-in sensors in the NHS Apps Library addressed the management of anxiety.

**Table 5:** Summary of health conditions where built-in mobile phone sensors were used.

Health condition	Curated mobile health app library				Total (n=18), n (%)
	NHS^a^ Apps Library (n=2)	AppScript (n=8)	MyHealthApps (n=8)		
Respiratory	0	4	0	4 (22)	
Dermatology and skin cancer	0	1	3	4 (22)	
Anxiety	2	0	1	3 (17)	
Neurology	0	1	2	3 (17)	
Visual acuity	0	2	0	2 (11)	
Audiology	0	0	1	1 (6)	
Cardiology	0	0	1	1 (6)	

^a^NHS: National Health Service.

### Sensor Types and Health Conditions

Mobile phone cameras are employed in addressing the broadest range of health issues (Table 6). For example, skin cancer assessment camera apps are available in MyHealthApps and AppScript repositories. Diagnostic pattern-matching algorithms analyze acquired camera images of skin lesions in one app in the MyHealthApps library, whereas another from AppScript captures photos for later analysis by a physician. General dermatology apps using smartphone cameras to capture images are listed in the MyHealthApps library. In addition, two apps for the assessment of tremor were identified in the AppScript and MyHealthApps libraries, using the touch screen to assess touch accuracy in Parkinson disease symptom assessment and accelerometer sensors to detect tremor-induced movements, respectively.

**Table 6:** Sensors, health conditions, and methodologies identified.

Sensor and health condition		Measure	Methodology used
**Camera**			
	General anxiety disorder	Heart rate	Photoplethysmography
	Cardiac	Heart rate	Photoplethysmography
	Dermatology (n=2)	Photography	Clinician inspection
	Respiratory (breathing exercise)	Heart rate variability	Photoplethysmography
	Skin cancer	Photography	Steganographic pattern matching from photo
	Skin cancer	Photography	Clinician inspection
**Touch screen**			
	Panic attacks	Screen image display	Images displayed to reduce panic
	Visual acuity (n=2)	Touch accuracy	Eye-hand coordination assessment and coaching
	Parkinson disease	Touch accuracy	Fine motor skill assessment and coaching
**Microphone**			
	Respiratory (sleep; n=2)	Snoring sound level and frequency	Snoring and apnea detection
	Respiratory (breathing exercise)	Breath sound detection	Feedback to encourage slow purposeful breaths
**Accelerometer**			
	Neurology	Tremor detection	Calculates tremor frequency
**Speaker and touch screen**			
	Audiology	Calibrated sound generation	Self-administered hearing test

## Discussion

### Principal Findings

Curation activities offered by third-party mHealth libraries, which are underpinned by medical device regulation, contribute to informing and protecting mHealth consumers. In this study, we surveyed three popular curated libraries regarding a specific subset of mHealth apps, namely, those using built-in mobile phone sensors for diagnosis or treatment of health conditions. Key aims of this survey included determining app availability, mobile phone operating system compatibility, intended purpose (diagnosis or therapy), types of sensors employed, and the range of health conditions where built-in smartphone sensors are used. First, this survey yielded a relatively small number of apps across the libraries examined, with differences found in the number of apps available between libraries. Second, more apps were available for the users of Apple iOS smartphones than for those of Android devices; cross-platform availability differed between the libraries surveyed. Third, the majority of apps offered treatment and combined diagnosis and treatment, with a smaller proportion offering dedicated diagnostic functionality. Fourth, cameras, touch screens, and microphones were the most frequently used mobile phone sensors in these apps. Finally, the range of health conditions addressed by these apps included respiratory, dermatological, anxiety, and neurological conditions.

### Finding Trusted Mobile Health Apps

Searching for apps related to particular health topics or medical concerns pose challenges for health professionals and consumers alike. Search engines, such as Google and Bing, which index available apps based on keyword search algorithms, often yield large volumes of uncurated search results for a given health topic [35,36,50,51]. Although the Apple and Google app stores categorize submitted apps for more focused searching (eg, *Health and well-being*), those searches can still return an overwhelming result list of indeterminate quality [4,35]. Search engines and app stores display star ratings and reviews to indicate the popularity of given apps, but these may not be reliable measures by which listed mHealth apps can be *trusted* [22,45]. In addition to high-level categorical grouping, third party–curated mHealth libraries offer more detailed subcategories and lists for specific health conditions and medical specialties.

No studies could be found that quantify the prevalence of mHealth apps using built-in sensors in curated mHealth app libraries. A 2016 review of health care–related apps available from the Google and Apple app stores identifies 80 clinical or health care–related mHealth apps for diagnosis or health monitoring [35]. Of the apps described in this review, mobile phone cameras are the most frequently employed sensor type, with camera images used by some apps for dermatological and ophthalmological diagnosis. Camera imaging is also employed in apps for blood flow monitoring by means of photoplethysmographic monitoring of pulse rate and estimation of blood pressure [35]. Emergent problems with blood pressure estimation received wide publicity when found to be unreliable in the case of at least one app [32]. In a number of health care contexts, app availability has been termed *volatile*, where apps may be removed from app stores in response to the revision of the underlying evidence base of an app or for medicolegal reasons [52].

Critics highlight a lack of transparency in standards applied to the screening of submitted apps before inclusion and hosting in popular app stores and search engines, resulting in mHealth app offerings, which may vary in quality or safety [7,45,51]. Measures of mHealth app quality have been developed (but not widely applied), including the (now defunct) Happtique Health App Certification, EU Kitemark certification, Intercontinental Medical Statistics (IMS) Score, and Mobile Application Rating Scale (MARS) [39,51]. For example, MARS evaluates mHealth app quality in five areas: aesthetics, functionality, engagement, information quality, and subjective quality [39,53]. Curated mHealth app libraries offer *trusted* sites for health consumers to select mHealth apps, constituting more detailed and specialized search portals than the aforementioned search engines and app stores [4,13]. Varying degrees of (proprietary) vetting are conducted to assert the safety and efficacy of curated apps, thereby imbuing search results with trust; detailed app scoring methodology and the incorporation of app quality measures, such as MARS, into the vetting process are not disclosed by these sites [40,54].

### Primary and Supporting Roles for Sensors

In contrast to a million health and well-being apps on offer to mobile phone users from popular app stores, only 18 mHealth apps using built-in smartphone sensors are identified in this survey, representing 1.50% (18/1200) of all mHealth apps collectively offered by the three curated libraries examined here (Table 1). A key consideration in the curation process is that medical device regulatory requirements may preclude listing of some apps in these libraries to prevent harm to app users [50]. Active mHealth apps (ie, those using sensors to gather health data) may be at greater risk of causing negative health impacts because of potential harm from inaccurate or incorrect data, demanding more rigorous curation and potential exclusion from curated libraries [23]. Regulatory authorities may require the assessment and accreditation of mHealth apps that offer diagnostic or therapeutic recommendations or those that transform the functionality of the mobile phone into that of a medical device [23,54]. Some sensor-based mHealth apps may use sensors as a secondary or supporting measure and thus not be regarded as medical devices per se [23]. Overall, two such examples are identified in this survey: anxiety and breathing exercise apps that use camera sensors for pulse detection as a secondary or indirect health data measure.

### Availability on Competing Mobile Phone Platforms

The respective smartphone market shares for Apple and Android devices are comparable [55,56]. In contrast, not all the apps identified in this survey are available across both popular mobile phone platforms, potentially disadvantaging some mHealth consumers. Half (9/18, 50%) of the apps identified in this survey are dedicated solely to Apple iOS, a further 11% (2/18) specific to Android, and only about one-third (7/18, 39%) available for both operating system platforms (Table 2). Apple iOS device manufacture is controlled solely by Apple, with relative homogeneity in hardware components such as sensors potentially offering app developers more stable or predictable target platforms for app development [56]. In contrast, Android devices may originate from multiple hardware vendors with disparate (sensor) hardware components, potentially adding complexity to the development of apps catering for a wider range of target device hardware and sensors [55,56].

Half of the identified mHealth apps (9/18, 50%) offer dedicated treatment features, with further about one-fourth (4/18, 22%) dedicated to diagnosis (Table 3). The imperative to seek (traditional doctor-patient) medical consultation regarding definitive diagnosis, potential risk of self-misdiagnosis, and regulatory restrictions may all contribute to the smaller proportion of purely diagnostic sensor-based apps offered by the libraries surveyed [57]. Dermatology and skin cancer diagnostic apps using the mobile phone camera constitute 4 of the 5 dedicated diagnostic apps identified.

### Health Concerns and Sensor Types

Cameras and touch screens are the most frequently used sensors in the identified apps, followed by microphones and accelerometers. Apps using camera sensors are most prominent in the MyHealthApps library, whereas AppScript lists more microphone and touch screen apps (Table 4). Notwithstanding contemporary research studies regarding the use of GPS for activity tracking in mental health conditions such as bipolar disorder and general depression, no examples of translating this research into GPS-based sensor apps were found in any of the libraries surveyed [20,26]. Anxiety therapy was the sole focus of both apps identified in the NHS Apps Library. Respiratory concerns were the most frequently addressed health conditions in the AppScript library, whereas apps related to dermatology and neurological conditions were more prevalent in the MyHealthApps library (Table 5). Cameras are employed in a wider range of health conditions compared with other sensors (Table 6). Photoplethysmography is used to measure heart rate and heart rate variability in three camera apps, whereas photo capture for later inspection by a clinician is offered by two dermatology and skin cancer diagnostic apps. A single camera app performs steganography (pattern matching) for skin cancer diagnosis.

### Conclusions

This survey found that mHealth apps using built-in sensors for diagnosis and treatment represented but a modicum of all apps found in the curated mHealth libraries examined. The nature and rigor of the curation process go some way to explain this observation, including the constraints of regulatory requirements for software deemed as medical devices. This may also help explain the smaller proportion of dedicated diagnostic apps observed in these libraries. Some health consumers may be disadvantaged by differences in the availability of apps on competing mobile phone platforms. Cameras, touch screens, and microphones were used most frequently in the surveyed apps. A limited range of health concerns were addressed by the surveyed apps.

Further efforts are needed to increase the availability of ubiquitous, low-cost mobile phone sensor technology in curated lists to assist with health conditions.

## Appendix

Multimedia Appendix 1 Details of mobile health apps using built-in mobile phone sensors in surveyed curated libraries.

## Abbreviations

MARS: Mobile Application Rating Scale
mHealth: mobile health
NHS: National Health Service
