# Supplementary material for: Assessment of Mobile Health Apps Using Built-In Smartphone Sensors for Diagnosis and Treatment: Systematic Survey of Apps Listed in International Curated Health App Libraries
Source: JMIR Mhealth Uhealth. 2020 Feb 3;8(2):e16741. doi: 10.2196/16741 (PMC7055743; doi:10.2196/16741)
Supplement: Multimedia Appendix 1 [file mhealth_v8i2e16741_app1.pdf]

This is a Multimedia Appendix to a full manuscript published in the J Med Internet Res. For full copyright and citation information see http://dx.doi.org/10.2196/jmir.16741

Details of mobile health apps using built-in mobile phone sensors in surveyed curated libraries.

Purpose	Sensor	App name	Description in library	Operating system	Health condition
**AppScript (n=8)**					
Diagnosis and treatment	Microphone	SnoreMonitor SleepLab	Graph your snoring and breathing sounds. Tap the graph to listen and analyze. Repeat tapping to accelerate playback speed.	iOS	Respiratory (snoring)
Diagnosis and treatment	Microphone	SnoreLab	SnoreLab records, measures, and tracks your snoring and helps you to discover effective ways to reduce it.	iOS	Respiratory (snoring)
Diagnosis and treatment	Microphone	Breathing Zone	Breathing Zone uses a clinically proven therapeutic breathing technique that decreases your heart rate and over time can even help lower high blood pressure.	iOS and Android	Respiratory (breathing)

Treatment	Camera	HeartRate+ Coherence	HeartRate+ includes a breathing guide and measures how well you are doing the breathing exercise via monitoring your heart rate variability.	iOS	Respiratory (breathing)
Diagnosis	Camera	SpotMole	This is the world’s first smartphone app to perform an automatic mole analysis using the device’s camera, gallery and embedded algorithms.	Android	Dermatology (skin cancer)
Treatment	Touch screen	Vision Training 1	Vision Training 1 is training for eye movement, eye-hand coordination, and binocular function.	iOS	Visual acuity
Treatment	Touch screen	Visual Attention Therapy Lite	Visual Attention Therapy Lite helps brain injury and stroke survivors, as well as struggling students, to improve scanning abilities.	iOS	Visual acuity

Treatment	Touch screen	pdFIT	Beneufit's pdFIT is a disease management tool for people with Parkinson disease. A finger tapping test allows users to objectively assess their manual dexterity and fine motor control on their own through the pdFIT app.	iOS	Neurology (Parkinson disease)
**MyHealthApps (n=8)**					
Treatment	Touch screen	Dexteria	App to improve fine motor skills as part recovering from injury or stroke or for learning development skills. Dexteria's hand and finger activities use the iOS multitouch screen to help build strength, control, and dexterity.	iOS and Android	Neurology
Diagnosis	Camera	iDoc24	Allows a user to send anonymous photos of any skin condition to a dermatologist.	iOS and Android	Dermatology

Diagnosis and treatment	Camera	MyPso	Helps people with psoriasis track their conditions and recording symptoms and severity as and when they happen.	iOS and Android	Dermatology
Diagnosis	Camera	UMSkinCheck	Allows a user to check for skin cancer by conducting self-examination.	iOS	Dermatology (skin cancer)
Treatment	Touch screen	Anti-stress Chromotherapy	Chromotherapy app intended to reduce mental stress by demonstrating the therapeutic value of 256 shades of color.	Android	Anxiety
Diagnosis and treatment	Camera	Instant Heart Rate	Measures heart rate and pulse. Uses the phone's camera to detect the pulse in a user's fingertip. The app beeps in time with the pulse.	iOS and Android	Cardiology
Diagnosis and treatment	Accelerometer	Lift Pulse	Records the extent of a user's tremor via the smartphone's accelerometer.	iOS and Android	Neurology
Diagnosis	Speaker	uHear	Self-administered hearing test.	iOS	Audiology
**NHS Apps Library (n=2)**					

Treatment	Touch screen	Beat Panic	The Beat Panic app uses a series of soothing colored flashcards with messages designed to help you overcome a panic attack in a calm, gentle manner.	iOS	Anxiety—panic attacks
Treatment	Camera	Chill Panda	Learn to relax, manage your worries, and improve your well-being with Chill Panda. The app measures your heart rate and suggests tasks to suit your state of mind.	iOS and Android	Anxiety—general
